# Supplementary material for: Patterns of Intron Gain and Loss in Fungi
Source: PLoS Biol. 2004 Nov 30;2(12):e422. doi: 10.1371/journal.pbio.0020422 (PMC532390; doi:10.1371/journal.pbio.0020422)
Supplement: Table S1 — Also available at http://genes.mit.edu/NielsenEtAl/. (4.3 MB ZIP). [file pbio.0020422.st001.zip › NielsenEtAl/html/165.html]

AN4616.1.NCU00692.1.MG02842.1.FG08644.1


```
 CLUSTAL W (1.82) Multiple Sequence Alignments - Introns Inserted


Sequence 1: NCU00692.1	571 aa
Sequence 2: MG02842.1	585 aa
Sequence 3: FG08644.1	561 aa
Sequence 4: AN4616.1	563 aa
Alignment Length: 591 aa
Number Identitical Residues: 269 aa
Alignment Score (without introns) 13422


MG02842.1 	MSENGTKGAVAPEDRVVIGITFGNSNSSIAYT-V~DDKAE0YLKRVHAKYSIDLTSKLAL
NCU00692.1	MSD--SKVSVAPSERTVIGITFGNSNSSIACT-V~DDKAE~VIANEDG------------
FG08644.1 	MSDNAGAKGPEPGDRVAIGITFGNSNSSIAYT-V~DDKAE~VIANEDG------------
AN4616.1  	MSDEINGTG----ERFAIGISFGNSSSSIARINP0EGKAE~VIANEEG------------
          	**:.    .    :* .***:****.****  .  :.***  : . ..            

MG02842.1 	PD~RQIPTILSYVDGEEYYGAQAKAFLVRNPKNTISYFRDFLGQD2FKSIDPTHCHASAH
NCU00692.1	-D1RQIPTILSYVDGDEYYGQQAKNFLVRNPKNTVAYFKDFLGKD~FAAVDPTHNHASAH
FG08644.1 	-D1RQIPTVLSYVDGDEYYGGQAKAFLVRNPENTIANFRDFLGQE2FKSIDPTHSHASAH
AN4616.1  	-D1RQIPSILSYIGGEQYHGTQAKAQLIRNPSNTVAYFRDYLGKD2FKSIDPTPCHNSAH
          	 * ****::***:.*::*:* ***  *:***.**:: *:*:**:: * ::***  * ***

MG02842.1 	PQEASDAVSFTVKDKEGDEESTVSVSEVATRYLKRLTGSASDYLGKKVTSAVITIPTNFS
NCU00692.1	PEDADGTVAFTVQEKEEGEASKLSVSEVSTRYLRRLIEAASEYLGKKVTSAVMTVPTNFS
FG08644.1 	PQEVSGSVAFTVKDKVEEDASSISVSEITTRYLQRLVGSASDYLGKKVTSAVVTVPTNFS
AN4616.1  	PIQSDSTVAFSIRDTESETPNTVTVSEITTRHLLRLKQSASDFLGREVNAAVVTVPTDFN
          	* : ..:*:*::::.     ..::***::**:* **  :**::**::*.:**:*:**:*.

MG02842.1 	DKQREALIAAANAADIEVLQLISEPTAAVLAYDAR-EQGVSDKIVVVADLGGTKSDVAVL
NCU00692.1	DKQKEALLAAAAAANLEVLQLINEPVAAALAYDARAETEVQDKIVVVADLGGTRSDVAVV
FG08644.1 	EKQREALIKSANDAGLEILQLISDPVAAVLAYDARPEAKVEDKIVVVADLGGTRSDVAVI
AN4616.1  	DAQREALTAAAKAAGLDILQLIHEPVAAALAYDARPEAVVTDKLIVVADLGGTRSDVAVI
          	: *:***  :*  *.:::**** :*.**.******.*  * **::********:*****:

MG02842.1 	ASRGGMYTILATAHDYEFAGVALDKVLMDHFAKEFMKRHAGVDPRSNAKSLAKLRLESES
NCU00692.1	ASRSGMYTILATVHDYDFHGTALDKVLIDHFSKEFIKKHQ-VDPRENARSLAKLKAEAES
FG08644.1 	ASRGGLYTILATAHDYEFAGVHLDNALVEHFAKEFQKKHN-IDPRNNARSLAKLRLESEA
AN4616.1  	ACRGGMYTILATAHDYELGGSTLDQIIIDHFAKEFIKKHK-TDPRENARGLAKLKMEGEA
          	*.*.*:******.***:: *  **: :::**:*** *:*   ***.**:.****: *.*:

MG02842.1 	TKKSLSLGSNASFSVESLAEGYDFSCTINRLRYETLARSVFEGINRLVESAVKKAGLDVL
NCU00692.1	TKKALSLSTNASFSVESLANGIDFTSSINRLRYETIARSVFEGFTRLVDSAVKKAGLDAL
FG08644.1 	TKRALSLGANAQFSVESLADGFDFSVTINRIRYEMVARKVFEGFNRLIEGVIKKAGLDVL
AN4616.1  	TRKALSLGTNATLSIESLTDGIDYGSTINRTRYELLSSKVFAQFTGLIEQVIKKAELDVL
          	*:::***.:** :*:***::* *:  :*** *** :: .**  :. *:: .:*** **.*

MG02842.1 	DVDEVILAGGSAHTPRIASNLGAIFPESTKILAPSTTPTAINPSELQARGAALQASLIQE
NCU00692.1	DIDEVILSGGTSHTPRIAANLGYLFPQTTKILAPSTTPAAINPSELNARGAALQASLIQE
FG08644.1 	DIDEVILTGGTSHTPRIANNLRTIFPETTEIQAPATSASAINPSELLARGAALQASLVQE
AN4616.1  	DIDEVIFSGGVSHTPKIAQLARNLFPEKTRILAPSTLASAINPSELSARGAAIQASLIQE
          	*:****::** :***:**     :**:.*.* **:* .:******* *****:****:**

MG02842.1 	YEADDIEQSTHPAVTTVKHITNAIGIVTL-EGDVENFTPVVAPETAVPARRIVRVAAPKD
NCU00692.1	YEAEDIDQSTHAAVTTVPHVTNAIGVISLNEAGEEVFVPVIAPETAVPARRTIHVAAPKD
FG08644.1 	YEASDIEQSTHPAVTTVKHISNAIGVITVGADGEDVFTPIVAPETAAPARRTVHIPAPKE
AN4616.1  	FETEDIEQSIHPMVTATPHLSKAIG-VEFTSGDAVEFLPLLNTETALPARRVAQYSVPAE
          	:*:.**:** *. **:. *:::*** : .   .   * *:: .*** ****  : ..* :

MG02842.1 	GGDVLIRVVEGGTHIKVTQPEPKPQKAKANGVQVEDAKESDDEDSDDSDEESEEDEEKRE
NCU00692.1	GGDVLVKIAEGNTHIKVTKPEPK-VKDESKAAKVEDADEEGD-DSDFSDDEEEEEEEKRE
FG08644.1 	GGDVLIKIAEGNTHIKVTKPEPK---------AKDEAKDEDD--SDFSDSD-EEDEETRE
AN4616.1  	GGDVLIRVCEGSREIKVTKPEPK--------PKEEKSKAEDDEDDSDFDSD-EEEEDIRE
          	*****::: **. .****:****           :.:. ..*....  *.: **:*: **

MG02842.1 	KVWKIGDSLAEAAIRGVKKGGQVEVTIQVMADLGVTVTAREVGAQGGVRGTL---
NCU00692.1	KVWKIGSTLAEAAVRGVKKGGKVEVTILVDANLSVTVTTREVGAKTGVRGTLSA-
FG08644.1 	KIWKIGSTLAEAAVKDVKAGGKVEVTINVLADLGVTVTTREVGGKGGVRGKI---
AN4616.1  	IVWKTEQPIAELAVKGVKAKGKVELMIHINADLGLQITAREVGGQSAVRGAVESP
          	 :**  ..:** *::.**  *:**: * : *:*.: :*:****.: .*** :.:.
```
